# Effects of Natural Disaster Trends: A Case Study for Expanding the Pre-Positioning Network of CARE International

**DOI:** 10.3390/ijerph9082863

**Published:** 2012-08-14

**Authors:** Melda Bozkurt, Serhan Duran

**Affiliations:** Department of Industrial Engineering, Middle East Technical University, 06800 Ankara, Turkey; Email: e159789@metu.edu.tr

**Keywords:** natural disaster trends, pre-positioning, humanitarian relief, location

## Abstract

The increasing number of natural disasters in the last decade necessitates the increase in capacity and agility while delivering humanitarian relief. A common logistics strategy used by humanitarian organizations to respond this need is the establishment of pre-positioning warehouse networks. In the pre-positioning strategy, critical relief inventories are located near the regions at which they will be needed in advance of the onset of the disaster. Therefore, pre-positioning reduces the response time by totally or partially eliminating the procurement phase and increasing the availability of relief items just after the disaster strikes. Once the pre-positioning warehouse locations are decided and warehouses on those locations become operational, they will be in use for a long time. Therefore, the chosen locations should be robust enough to enable extensions, and to cope with changing trends in disaster types, locations and magnitudes. In this study, we analyze the effects of natural disaster trends on the expansion plan of pre-positioning warehouse network implemented by CARE International. We utilize a facility location model to identify the additional warehouse location(s) for relief items to be stored as an extension of the current warehouse network operated by CARE International, considering changing natural disaster trends observed over the past three decades.

## 1. Introduction

The increased number of natural disasters and intensified damage they have caused in recent years necessitate better coordination and planning in delivering humanitarian relief. Unlike commercial logistics, in the humanitarian case demand is highly unpredictable. Thus, it becomes crucial to focus on developing management strategies to increase the effectiveness and minimize the response time of the warehouses supporting relief operations. Pre-positioning is such a strategic attempt that allows faster response and moreover better procurement planning [1].

Wassenhove defines pre-positioning as a tool enabling to respond faster by locating items like medical supplies and food in warehouses close to the disaster-prone regions [2]. UNICEF is actively utilizing pre-positioning relief supplies in warehouses near the affected areas and reports this strategy to be effective in responding to emergencies immediately, although not sufficient to cover all the needs [3]. Similarly, some of the major humanitarian Non-Governmental Organizations (NGOs) have also constructed their own pre-positioning warehouse networks. World Vision International (WVI) implements a pre-positioning system which is based on Global Pre-positioning Units (GPUs) [4]. The GPU system was launched with three warehouse locations in 2000: Denver, Colorado; Brindisi, Italy and Hanover, Germany. 

The establishment of the pre-positioning network requires the following two key decisions to be given; determining warehouse locations and deciding inventory allocations among those locations [5]. Modeling these decision problems by mathematical programming methods and solving it using Operations Research (OR) techniques is an appropriate approach [6,7]. The locations of the pre-positioning warehouses can be found using p-median, p-center and covering models [8]. Akkihal, using a p-median model, compared humanitarian and military operations and concluded that the requirements of both of them are similar as material demands are often unexpected and rapid response is critical to saving lives [9]. To incorporate the uncertainty in disaster magnitude, type and location, the mathematical models in facility location literature are modified to consider different demand scenarios. Balçık and Beamon constructed a model to determine the locations and the number of pre-positioning warehouses to maximize the total expected demand covered considering a set of scenarios [10]. Their model make inventory decisions with a budget limit and in the presence of acquiring, storing and shipping costs of the relief items, whereas the one in Duran *et al.* [11] gives inventory decisions without a budget constraint since the locations suggested by the non-profit organization they collaborate (CARE International), are no- or low-cost locations. The recent work by Apte provides a comprehensive literature review on facility location models used in humanitarian logistics [12].

Although the location models used in humanitarian logistics are modified versions of the models in literature capturing the uncertainty in demand, additional care is needed while using them. Once the pre-positioning warehouse locations are established, they will be utilized for a long time. Therefore, the chosen locations should be robust enough to enable extensions, and cope with changing disaster trends in disaster types, locations and magnitudes. Consequently, trend analysis may be crucial to determine the optimal locations for pre-positioning warehouses. Unfortunately, the trend in the number of affected people is influenced by major events that have high impact. The number of affected people and natural disaster occurrences change considerably from year to year. According to the Annual Disaster Statistical Review the year 2006 had less human impacts compared to the recent years [13]. The number of deaths by natural disasters increased from 23,000 casualties in 2006 to 235,000 in 2008 [14]. These facts call for the study of longer time periods to detect a possible trend such as an increasing trend of disaster occurrences among different decades. 

In this study, we use decade aggregation level to analyze the disaster trends to overcome annual fluctuations. We utilize Emergency Events Database (EM-DAT) to obtain the disaster data which includes data on the effects of disasters all around the world from 1900 to the present [15]. The data contains locations, dates, number of affected people, duration of each disaster and disaster-related economic damage estimates. We use the data between the years 1977 and 2006 and divide this time period into three decades to observe whether there is a detectable change in the locations of disasters and the number of affected people due to those emergencies. Finally, we use the years 2007, 2008, 2009 and the first six months of 2010 to verify our findings. 

## 2. Natural Disaster Statistics

Between the years of 1977 and 2006, 4,326 natural disasters occurred and 3.5 billion people were affected during those thirty years. From Table 1 we observe that highest number of disasters occurred in the South Eastern Asia, Southern Asia and Eastern Asia regions, in the mentioned order, but the statistics we are really interested in are on how those disaster-related statistics change from one decade to another when the 1977–2006 time horizon is divided into three decades.

**Table 1:** Natural Disasters and Affected People by Regions (1977–2006).

Number of Disasters Occurred			Number of Affected People			Region
1977–1986	1987–1996	1997–2006	1977–1986	1987–1996	1997–2006	
34	61	164	1,248,151	1,199,263	10,185,405	Northern America
46	74	124	9,484,275	2,866,447	11,909,294	Central America
97	101	174	19,654,312	11,668,911	9,078,576	South America
11	19	59	31,694	409,390	4,081,350	Western Europe
2	8	23	18	1,001,080	286,281	Northern Europe
41	26	79	2,621,127	111,862	1,369,287	Southern Europe
10	27	115	416,399	3,262,711	5,099,714	Eastern Europe
19	27	76	2,660,387	3,468,531	6,138,633	Western Asia
0	10	29	-	542,414	604,004	Central Asia
145	184	295	276,135,472	467,522,586	393,341,107	Southern Asia
111	171	313	22,938,574	906,987,392	1,004,086,931	Eastern Asia
174	201	276	54,937,829	72,234,216	71,901,737	South Eastern Asia
14	28	84	1,116,030	2,571,369	2,918,931	Western Africa
16	26	49	1,337,983	3,207,891	4,383,401	Northern Africa
3	14	44	2,450	396,363	1,279,755	Middle Africa
6	15	36	1,213,885	223,131	661,494	Southern Africa
40	51	172	4,040,294	6,516,999	19,498,878	Eastern Africa
32	69	90	3,590,680	6,847,385	11,807,417	Caribbean
19	34	49	28,402	3,986,725	53,679	Australia and New Zealand
7	10	11	172,574	291,005	49,114	Polynesia
19	18	31	948,146	536,479	210,632	Melanesia
0	5	8	-	12,318	30,695	Micronesia
846	1179	2301	402,578,682	1,495,864,468	1,558,976,315	**TOTAL**

Within the years of 1977 and 1986, 846 natural disasters occurred and 402 million people were affected. The highest number of disasters within a region occurred in the order of South Eastern Asia, Southern Asia and Eastern Asia regions. The highest number of affected people by regions can be listed in the order of Southern Asia, South Eastern and Eastern Asia. When we compare the data from years between 1987 and 1996 to the prior 10 years’ period, we observe that the number of natural disasters increased to 1179, while the number of affected people increased more than triple-fold and became 1,495 billion. Like in the previous decade, South Eastern Asia was the region that suffered the largest number of disasters. Southern Asia and Eastern Asia regions follow as the second and third. Eastern Asia region topped the list of the highest number of people affected and Southern Asia and South Eastern Asia regions follow Eastern Asia in this category. Unfortunately, the number of disaster occurrences continues to follow this increasing trend with 2,301 disasters in the period of 1997–2006 resulting in 1,558 billion affected people.

Analysis of the data of a thirty-year period shows an increasing trend in the number of natural disasters (from 846 to 2,301) and in the number of people affected (from approximately 400 million to 1.5 billion). In the first decade Eastern Asia ranked third place for both categories, while in the second and third decades the highest number of affected people belonged to the Eastern Asia region. In all regions of Africa except Southern and in the regions of Caribbean, Western Asia, Eastern Asia, Central Asia, Eastern Europe, Western Europe, and Micronesia, we observe an increasing trend in the number of natural disasters and affected people, whereas in the Melanesia and South America regions we observe a decreasing trend in the number of people affected. 

Although the trends among decades and regions for these two main disaster-related statistics are easy to observe, there are many other factors affecting the efficiency of a pre-positioning network such as the time gap between disasters, the dispersion of disaster locations and the socio-economic structure within the regions. Therefore, a sophisticated model is required to be used if we want to design a robust pre-positioning network that takes the disaster occurrence trends into account. 

## 3. A Case Study for Expanding the Pre-Positioning Network of CARE International

CARE International is one of the largest private international humanitarian aid organizations in the World with programs to help the people in need. Although CARE mainly aims at long-term development, it also supports emergency operations in more than 69 countries worldwide [16]. 

### 3.1. Current Prepositioning Network of CARE International

As a result of the collaboration between CARE and Georgia Tech started at the beginning of 2007, CARE established a pre-positioning network of three warehouses between the years of 2008 and 2010; a first warehouse in Dubai, the second in Panama and the last one in Cambodia. This pre-positioning network is designed with the help of the location model developed by Duran *et al.* [11] which will also be used in this study. 

The model considers demand for relief supplies caused by sudden-onset natural disasters; earthquakes, windstorms (hurricanes, cyclones, storms, tornadoes, tropical storms, and typhoons) and floods. The center of population of the United Nations’ 22 sub-regions are used as the demand locations. Relief items are preferably supplied from the inventory located at the pre-positioning warehouses that are opened among the candidate locations. Possible warehouse locations are indicated by CARE International as: Cambodia, Hong Kong, Denmark, Germany, Honduras, India, Italy, Kenya, Panama, South Africa, Dubai and Miami. In the case of inventory shortage, global suppliers send relief items to the beneficiary regions directly with a long leadtime. 

Apparently, total inventory level and the number of warehouses to open are the main parameters affecting the performance of a pre-positioning network. Thus, these are the main parameters of the location model in Duran *et al.* [11]. Given these two main parameter levels, the model will minimize or maximize an objective by determining the locations of the warehouses to open. Being a non-profit humanitarian organization, CARE International enjoys low warehouse operating costs due to supports from governments and other organizations such as The United Nations Humanitarian Response Depot (UNHRD) [17]. Therefore, the objective is minimizing the weighted average response time of the pre-positioning network to the disasters that happen within a two-week time horizon for different demand instances created from the EM-DAT database. Two-week leadtime estimation is based on the experience of CARE International with its suppliers. Most of the global suppliers were reported by CARE to send the relief items within two weeks [11]. In the model, it is assumed that the demand can be met and warehouses can be replenished by these global suppliers. 

The model was executed for only ten years’ time period (1997–2006) in Duran *et al.* [11]. In this study, we enhance the findings by executing the model for the past thirty years to analyze the effects of natural disaster trends on the pre-positioning network implementation and expansion. We also verify our results using the natural disaster data for the years 2007, 2008, 2009 and 2010.

### 3.2. Consideration of Natural Disaster Trends

We first divide the demand data we obtain from EM-DAT database for the years of 1977–2006 into three decades. Years prior to 1977 are omitted from the study due to poor data quality and consistency. Since during the 2-week replenishment period of the warehouses multiple disasters may need response from the pre-positioning network to be designed, *demand instances* have to be created and used as input for the mathematical model for each decade to configure the best network structure. To accomplish this disaster data of each decade is sorted according to each disaster’s start date. Utilizing the time between two disaster occurrences, disasters are grouped into demand instances including all disasters that occurred in two-week time periods. Each demand instance consists of demand quantities for different relief items at one or more demand points. 

The calculation of the total number of relief items in a demand instance considers the types and locations of disasters (each disaster type may require different kind of relief items when it hits a different region of the world) and the amount of the relief item required per affected person by that disaster. In the EM-DAT database, “affected” is defined as “requiring basic survival needs such as food, water, shelter, sanitation *etc…*” and we consider seven relief items; cold tent, hot tent, household utensils, medical relief items, hygiene sets, sanitation sets and water. The amount of relief items required by a person is derived from CARE International’s specifications [16]. In particular, CARE specifications state that one tent should be provided for five people, one medical equipment kit can supply 50, three liters of water are required daily per person. Finally one hygiene item kit is adequate for the needs of eight beneficiaries. Also as illustrated in Table 2, we adapted the likelihood (low, medium, high) of the need data from the International Federation of Red Cross and Red Crescent Societies (IFRC) for each relief item by different disaster types [18]. Moreover, considering the location of the disaster the number of the relief items required at a demand location is calculated. 

**Table 2:** Likelihood of Need of Relief Items.

	Earthquakes	Floods
**Water and sanitation**		
Distribution, storage, processing	**H**	**H**
Personal hygiene	**H**	**M**
**Shelter and household stock**		
Emergency Shelter	**L**	**L**
Kitchen utensils	**H**	**M**

The Mixed Integer Programming (MIP) model developed in Duran *et al.* [11] for given two main parameters; the number of warehouses to open (changes in a range of 1–9) and the inventory level to be rationed between those warehouse (high, medium and low) evaluates the possible pre-positioning warehouse locations using the demand instances for a given decade. The model chooses the pre-positioning warehouse network that minimizes the expected average response time over all demand instances assuming that each demand instance has the same probability to occur. The response time is defined as the time required for the initial shipment to arrive at an entry port of the affected region. If relief items can be sent from a warehouse in the network, the response time is equal to the fly time of the great arc distance between the warehouse and the demand point at the speed of a common cargo airplane plus one day for preparation at the warehouse. Otherwise, *i.e*., when pre-positioning network inventory is not sufficient to meet the demand, the unsatisfied demand is addressed by the global suppliers with a response time of two-weeks. For a specific demand instance, average response time of the pre-positioning network is therefore calculated as the weighted times of these two response times, weights corresponding to the proportions of demand met from each source. 

Analyzing three decades separately, obtaining the pre-positioning networks for each decade and comparing the change of warehouse locations among these three decades will enable us to observe the direction where the probability of disaster occurrence drifts towards indirectly. Including disaster trend analysis into the study ensures a robust pre-positioning network and a more confident expansion plan for the future. 

### 3.3. Computational Study

We solve the MIP model several times to obtain 27 different solutions for each decade as we try all the combinations for 9 candidate warehouses to open and three inventory levels: high, medium and low (corresponding to 100%, 50% and 25% of the average demand per demand instance). We performed the computations for each ten-year period using a high-level modeling software for mathematical programming and optimization (GAMS with the CPLEX solver). The model includes 22 demand points, 12 candidate warehouse locations, seven relief items and different numbers of demand instances for each period created from historical data of that ten-year period. 

**Figure 1:**
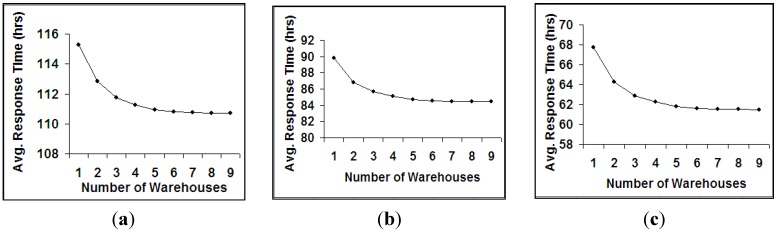
Average Response Time under Low, Medium, and High Inventory Levels for 1977–1986 Time Period. (**a**) Low Inventory Level; (**b**) Medium Inventory Level; (**c**) High Inventory Level.

Although the costs associated with opening and operating warehouses are low for CARE International, opening many pre-positioning warehouses are not preferable due to the management and the coordination difficulties. To find the best number of warehouses to open, the pre-positioning network is expanded by adding new warehouses one by one. The average response time of the pre-positioning network is observed to decrease for a specified level of inventory at a diminishing rate and later, the marginal benefit from opening an additional warehouse reaches a minimum. We observe that optimal number of warehouses to open fluctuates between 3 and 4 according to the inventory level for each decade. Figure 1 illustrates this observation for the 1977–1986 time period. 

We first focus on three-warehouse network to detect the disaster trends. Low inventory level is preferred while determining the best possible locations for pre-positioning relief items due to two reasons. First of all some of the relief items are perishable and the inventory holding cost is high. Secondly, it is not easy for an humanitarian organization to raise funds for purchasing relief items to pre-position. Donations and funds are mostly received after disaster hits a region and gets media coverage and they are also tagged to be used only at responding to a specific disaster.

**Figure 2:**
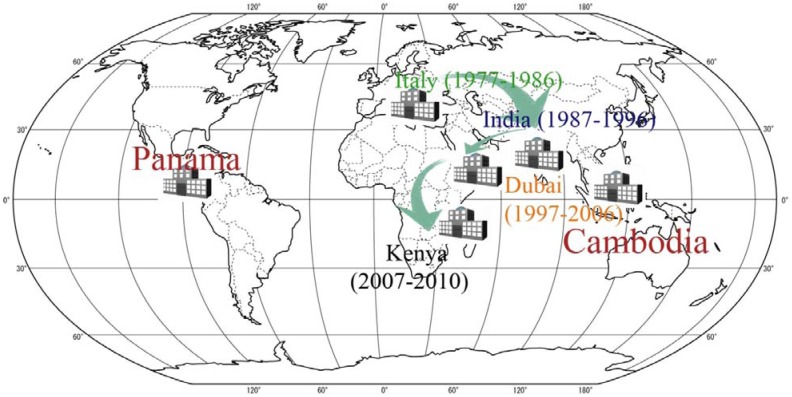
The change in location of the third warehouse with respect to decades.

When the results obtained from the computational study are analyzed: Cambodia, Italy and Panama are the optimal locations for 1977–1986; Italy is replaced by India in the 1987–1996 time period and Cambodia is replaced by Hong Kong and India is replaced by Dubai in the last decade of 1997–2006. Finally the best three warehouse locations provided by the model are Panama, Kenya and Cambodia for 2007–2010. While Panama and Cambodia (or Hong Kong which is considerably close to Cambodia) appear in almost every result, the location of the third warehouse indicates a clear shift. The change in location of the third warehouse from Europe to Africa is illustrated in Figure 2.

**Figure 3:**
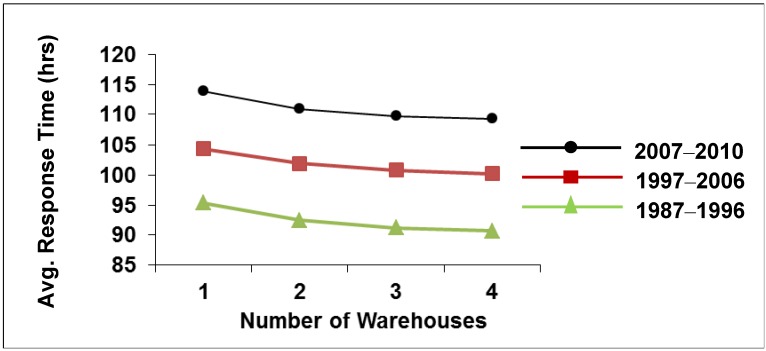
The change in Average Emergency Response Time.

The average response times of the optimally designed three-warehouse networks are 112, 91, 101 and 110 hours for the time periods of 1977–1986, 1987–1996, 1997–2006 and 2007–2010, respectively. After the first decade, as seen in Figure 3, the average response time of the network is getting higher although the locations are chosen optimally for each time period and the inventory levels are 25% (low) of the average demand per demand instance in each time period. There are two possible approaches to decrease the response time, which are directly concerned with the financial support nature of non-profit organizations. One of them is increasing the inventory level to pre-position and the other is opening a new warehouse.

The candidate locations considered are no- or low-cost warehouses or warehouse spaces that are available to CARE International through either governmental donations or UNHRD Network. Thus, these warehouse spaces are limited and cannot hold too much inventory on hand as relief items. Consequently, increasing the level of inventory to hold in a warehouse is not practical to reduce the average response time, considering the fact that minimum average inventory level among the demand instances we used is approximately 5 million. Therefore, we proceed with the idea of expanding the pre-positioning warehouse network by increasing the number of the warehouses. 

### 3.4. Optimal Four-Warehouse Network

Up to this point, our goal has been to find the best possible locations to pre-position relief items by considering the average emergency response time from each candidate location. The locations differ from decade to decade as shown in Figure 2. While interpreting the meaning of these location changes, we should note that the inventory levels used for each decade are determined from that decade’s average demand requirements. Therefore, if only the number of affected people and/or the number of disasters occurred were increasing proportionately (in other words, if only the number and/or impact of disasters hitting each region is increased with the same proportion); the best possible warehouse locations should be the same or change inconsiderably. However, it is verified that two locations stay almost the same (first location being Panama and second one Cambodia or Hong Kong which are considerably close to each other) for all decades and the place of the third warehouse location shifts from South Europe to Eastern Africa within 33 years. 

The existing locations that are in use today (found from 1997–2006 time period)—Panama, Hong Kong and Dubai—do not meet the requirements as the disaster trend is shifting to Eastern Africa. Moreover increasing emergency response time values in Figure 3 from decade to decade necessitates the investigation of including a fourth warehouse into the pre-positioning network. Additional warehouses can be opened with low levels of financial support since the locations are no- or low-cost locations provided by government and/or UNHRD.

**Figure 4:**
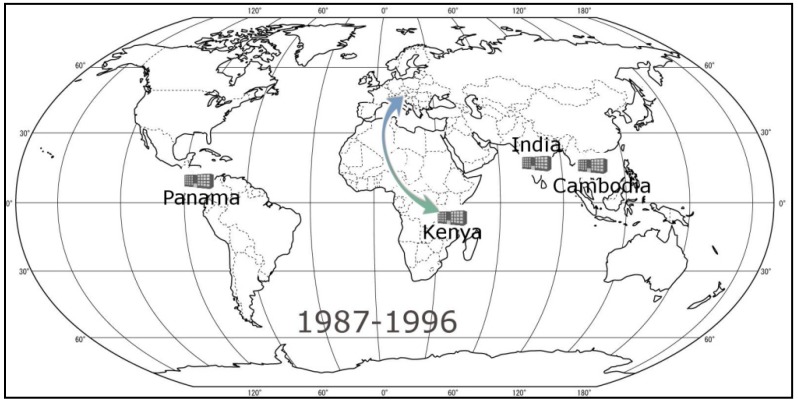
The change in fourth warehouse location from 1977–1986 period to 1987–1996 period.

We solve the model again for three decades and the most recent four years data by considering low inventory level and four warehouses to open. For the first decade (1977–1986), low inventory level is 1,200,000 items. As a result of solving the model for four warehouses to open, India becomes the fourth warehouse location in addition to the three-warehouse network of Panama-Italy-Cambodia found in Section 3.2. For the second decade (1987–1996), low inventory level is 4,500,000 items which is more than triple that of the first decade. The fourth warehouse location is determined to be Kenya as an addition to previously found Panama-India-Cambodia network. As seen in Figure 4, the fourth warehouse shifts to Eastern Africa from Europe from the first decade to the second one. The last decade (1997–2006), also refers the current situation for three warehouse network of Panama-Dubai-Hong Kong, is suggested to be transformed to Panama-Kenya-India-Hong Kong network by the model which is close to the network structure shown in Figure 4. 

**Figure 5:**
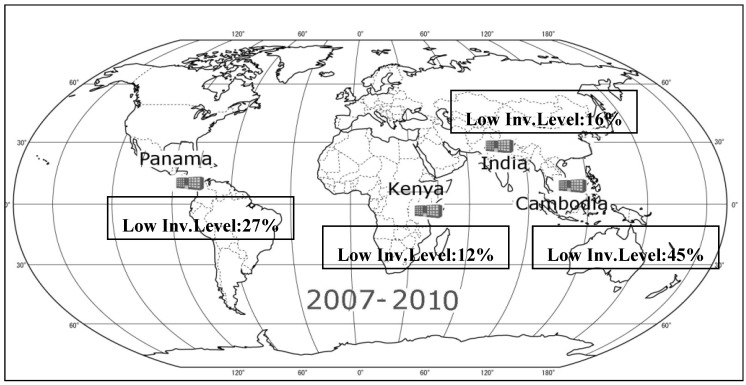
Optimal four warehouse network with inventory dispersion calculated from 2007–2010 time period.

When we apply the model for the years of 2006–2010, the four warehouse locations were found again as Cambodia, Kenya, Panama and India. Therefore, we conclude that the optimal four-warehouse network should be Panama, Kenya, India and Cambodia with 27%, 12%, 16% and 45% of the total inventory held in these locations, respectively, which is illustrated in Figure 5. 

The average emergency response times of the four-warehouse networks are observed to be lower (approximately half an hour) than the three-warehouse networks although the total inventory level held in the network does not increase. 

### 3.5. Expansion Plan

Kenya appears as a good candidate for the fourth warehouse location since Kenya is one of the optimal locations for the most recent three-warehouse network (2006–2010) and is optimal for nearly all four-warehouse networks for the time periods studied so far. When we consider the current three warehouse configuration situation; 17% of the inventories are suggested to be located in Panama, 48% of the inventories in Hong Kong and 35% of inventories in Dubai [11]. Therefore, in compliance with our findings, we suggest a new inventory distribution for the pre-positioning network. Simply opening a new warehouse in Kenya, and moving half of the inventories in Dubai warehouse to Kenya will result in a four-warehouse network which will operate with an efficiency close to the optimal network structure of Cambodia, Kenya, Panama and India found in Section 3.4.

## 4. Conclusions

In this study, we focus on the change in natural disaster trends throughout decades to avoid an ill-designed humanitarian pre-positioning warehouse that will be in place for many years. In order to analyze the effects of disaster trends on pre-positioning network development and expansion, we choose the case of CARE International which recently implemented and is operating a three-warehouse network. To identify the possible trends, first we collect the relevant disaster data and group them into two-week periods to create demand instances. Each demand instance of a certain decade may include several disasters hitting different regions of the world and the network has to respond to them simultaneously without replenishment. 

The next step was determining the optimal locations to open the pre-positioning warehouses considering the demand instances of that decade. A location model developed by Duran *et al.* [11] is utilized for that purpose. The model is executed for different number of warehouses to open (1–9) and inventory levels (low, medium and high) for each decade separately. We analyze the results by focusing on the low inventory level case since pre-positioned inventory cost is hard to finance for NGOs. 

First, we considered three warehouses to open for the low inventory level to observe the change in warehouse locations for pre-positioning relief items. It is seen that while Panama and Cambodia were stable locations for all decades the location of the third warehouse changed in each decade. In the first decade, the location was found as Italy, it was replaced by India in the second decade and Dubai in the last decade. These findings show that there is a possible trend in natural disasters; indicating a shift towards South. We used the data of the years 2007, 2008, 2009 and 2010 to verify the shift of the natural disaster trends. The result for recent years verified the natural disasters trend shifting to South Africa as the third warehouse location was found as Kenya. This observation suggests that the current locations decided in 2007 for CARE International may not be optimal anymore. 

Moreover, we also observe that average emergency response times of the optimal three-warehouse networks are increasing decade by decade. Considering the funding scheme of CARE International, opening an additional warehouse option is selected to improve the performance of the network as the average emergency response time decreases with the number of warehouses. Analyzing the optimal four-warehouse pre-positioning network for the same 33 years’ time period with the same methodology, an expansion plan considering the natural disaster trends is suggested.
